# The Trade-Off Between the Increased Colony Nurturing Ability and the Decreased Lifespan of Worker Bees (*Apis mellifera*)

**DOI:** 10.3390/insects16060558

**Published:** 2025-05-24

**Authors:** Chaoxia Sun, Hongji Huang, Mei Yang, Guoshuai Ma, Xinyao Huang, Shaokang Huang, Xinle Duan, Jianghong Li

**Affiliations:** 1 College of Bee Science and Biomedicine, Fujian Agriculture and Forestry University, Fuzhou 350002, China; 2 Fujian Honey Bee Biology Observation Station, Ministry of Agriculture and Rural Affairs, Fuzhou 350002, China

**Keywords:** colony temperature, adult bees, longevity, nurturing capability, lipid metabolism

## Abstract

High temperature is normally harmful to an organism. However, honey bees evolve, maintaining a relatively higher colony temperature of 34.5 °C in the long reproduction period. To determine the effect of such a higher colony temperature on adult bees and its biological significance, newly emerged bees were reared in cages at 34.5 °C and room temperature of 25.0 °C, respectively. Their survival rate, head weight, royal jelly-secreting gene expression, and morphology of the hypopharyngeal gland were investigated. Moreover, 40-day-old bees with significant differences in survival rate between the two temperature groups were subject to transcriptome and lipidome analysis. The result showed that the higher colony temperature was overall negative for the bees’ longevity. Transcriptome analysis showed that fatty acid metabolism-related items were enriched and the involved genes were upregulated in honey bees reared at 34.5 °C compared with the honey bees reared at 25.0 °C. Lipidomic analysis further validated that fatty acid metabolism, especially sphingolipid metabolism, was significantly altered. Such upregulation of fatty acid metabolism-related genes was also detected in young adult bees of 5 days old reared at 34.5 °C. These bees had heavier head weights, higher expression of royal jelly-secreting-related genes, and more developed hypopharyngeal glands. Such results showed that the colony temperature of 34.5 °C could accelerate the development process of newly emerged bees to be nurse bees, significantly increasing the colony nurturing capability, which in turn increased the development speed, size, and survivability of the colony. Thereby, the colony temperature of 34.5 °C shortened the lifespan of individual bees, but obtained huge returns at the colony level, with remarkable biological significance.

## Simple Summary

Honey bee colonies strictly keep a stable temperature of 34.5 °C when broods are reared in reproduction season. All the adult bees in the colony must live their lives under such temperature. Many reports have shown that a high temperature has a universal negative effect on a variety of animals. Therefore, the effect of such a high colony temperature of 34.5 °C on adult bees and its biological significance are scientifically extremely important. This study found that, like other animals, the colony temperature could shorten the lifespan of adult bees when compared to bees kept at room temperature of 25.0 °C. However, the upregulation of fatty acid metabolism-related genes caused by the higher colony temperature could quicken the development of young adult bees, increasing their royal jelly-secreting ability and whole colony nurturing capability. However, the colony nurturing capability fundamentally determined the development speed, scale, and ultimate survivability and competition of the colony. As a eusocial insect, the evolution of colony temperature primarily focuses on colony-level requirements. Honey bees make an optimistic trade-off between colony benefits and individual worker bee’s requirements.

## 1. Introduction

Temperature is one of the most important environmental factors affecting the development, physiological state, behavior, and population distribution of animals [1,2]. Temperatures in different seasons and topographic locations are different and annually changed, which brings stress to the lives of a variety of animals [3,4]. Accordingly, animals evolve many mechanisms to cope with the stress of temperature for their survival [5]. Based on the method of maintaining body temperature, animals are divided into homeotherms, keeping a constant internal temperature regardless of changes in the surrounding environment, which are better equipped to colonize diverse habitats including extreme environments, and poikilotherms, with a body temperature changing with the external temperature, which is more energy-efficient and less reliant on external resources and can allow growth in environments with limited food availability [4,6].

Longevity is a comprehensive reflection of animal fitness. Temperature is an important modulator of aging in both poikilotherms and homeotherm animals and affects their life history traits [7,8,9,10]. Studies on multiple species have linked higher temperatures with shorter lifespans and lower temperatures with longer lifespans in both poikilotherms and homeotherms [11,12,13,14,15,16,17,18]. Moreover, caloric restriction could extend the longevity of multiple animals by reducing body temperature, and low body temperature could improve health and longevity independent of caloric restriction [19].

The effect of temperature on longevity is traditionally explained using the rate-of-living theory, which posits that high temperatures increase chemical reaction rates and free radical production, thus speeding up the aging process [20,21]. However, further research showed that it is not simply a passive thermodynamic phenomenon, but rather an active process regulated by genes, especially those encoding thermosensitive TRP channels [22,23,24]. The regulating process is also involved in the systemic regulation of cytosolic proteostasis [25], sterol endocrine signaling, lipid homeostasis, the unsaturation level of fatty acid, germline-mediated prostaglandin signaling, autophagy, superoxide dismutase and phospholipase A2 activity, free radicals and their mediated lipid peroxidation, and co-chaperone p23, etc. [13,16,20,21,26].

With the increase in world economic activity, greenhouse gases such as carbon dioxide have been continuously released for decades, which has significantly increased the global temperature and caused climate warming [27,28]. Such global warming causes the frequent occurrence of extreme weather and the outbreak of pests or death of beneficial organisms due to the difference in adaption to temperature changes, which might disrupt the ecological balance and ultimately threaten the survival of human beings [29,30,31,32,33]. Studies of the response and adaption of animals to temperature stress would provide a basis for protecting beneficial organisms and avoiding pest damage under such a global warming background [34].

The honey bee is a typical poikilotherms insect, with body temperature affected by the external temperature. Its foraging activity is largely affected by the ambient temperature [35,36]. However, as a typical eusocial insect, they have evolved a surprising capability to accurately maintain a stable colony temperature at 34.5 ± 1.5 °C in the larval rearing period, regardless of the external temperature and its variation [37,38,39]. This provides the poikilothermic honey bees at the individual level with the traits of homeotherms at colony levels. The broods achieve optimal development conditions in the colony. However, the adult bees in the colony are inevitably affected by the relatively higher colony temperature, which might negatively affect the fitness of adult bees. However, the specific effect of colony temperature on adult bees, their response, and its biological significance has not been fully determined so far.

In a colony, each bee performs its own work and the whole colony runs in an orderly way. A colony with a brood always keeps the temperature at 34.5 °C [37,38,39]. To determine the effect of temperature on adult bees and its biological significance, newly emerged bees were reared in cages at 34.5 °C and room temperature of 25 °C, respectively. The survival rate, development of the hypopharyngeal gland, transcriptome, and lipidome of honey bees were investigated. The result showed that high temperatures could accelerate the development of newly emerged bees to become royal jelly-secreting nursing bees, which promotes the colony nursing capability and survivability, but impairs their longevity. There was a trade-off between the shortened lifespan of adult bees and increased colony nurturing ability regulated by lipid metabolism at the high colony temperature. The results contribute to our understanding of honey bee biology regarding temperature and pave the way for related research and applications.

## 2. Materials and Methods

### 2.1. Honey Bees

Honey bees (*Apis mellifera*) were collected from the colonies kept at the apiary of the College of Bee Science and Biomedicine, Fujian Agriculture and Forestry University, Fuzhou, China. The test was performed in May 2024, when the colony was nursing many broods. In detail, the combs with emerging bees from the colonies without apparent diseases, after removing the adult bees, were brought to the lab and put in an incubator (LHS-150HC-I, Yiheng, Shanghai, China) at 34.5 °C, 75% RH. Newly emerged bees less than 12 h old were collected. In total, 12 cages each with 60 newly emerged bees were set up and equally divided into two groups. In total, 6 cages were put in an incubator at a colony temperature (CT) of 34.5 °C, 75% RH, and another 6 cages were put in an incubator at room temperature (RT) of 25.0 °C, 75% RH. A piece of pollen pellet and 50% sugar solution (*w*/*v*) in a syringe were provided for each cage. Food was renewed every two days. The dead bees in each cage were counted daily for survivor analysis. The sugar solution consumed per cage was weighed daily. The daily sugar consumption per bee was obtained through dividing the sugar consumption per cage by the number of bees in the cages. Twenty heads of 1-day-old bees to 10-day-old bees and 20-day-old bees, respectively, from the two groups were weighed using an analytic balance (XPR226DRQ, Mettler Toledo, Shanghai, China). Hypopharyngeal glands were dissected from the head and observed under a microscope (DM2000 LED, Leica microsystems CMS Gmbh, Wetzlar, Germany).

### 2.2. Transcriptome Analysis

The survival rates of 40-day-old bees from the two temperature groups showed the most significant difference. Therefore, these bees were chosen for transcriptome analysis to explore the difference in gene expression. In detail, 10 bees from one cage were pooled as one mix sample, and three repeated samples from the CT group and RT group, respectively, were prepared and sent to Sangon Biotech, Shanghai, China, for transcriptome sequencing and subsequent analysis. Raw reads produced by sequencing were treated using Trimmomatic (Version 0.36) to obtain the clean reads, which were further blasted with the reference genome of *Apis mellifera* (https://ftp.ncbi.nlm.nih.gov/genomes/all/GCF/000/002/195/GCF_000002195.4_Amel_4.5/GCF_000002195.4_Amel_4.5_genomic.gff.gz, accessed on 2 April 2025) using HISAT2 (Version 2.1.0). TPM (Transcripts Per Million) was used to evaluate the gene expression level using StringTie (Version 1.3.3b). DESeq (Version 1.26.0) was used to screen the differently expressed genes (DEGs) between the two groups of samples with a significance level of qValue (adjusted *p*-value) < 0.05 and |FoldChange| > 2. Clustering of these DEGs was performed using the gplots package of R (Version 2.17.0). GO and KOG enrichment were performed using clusterProfiler (Version 3.0.5) with a significance level of qValue < 0.05.

### 2.3. LC/MS Non-Targeted Lipidome Analysis

To further validate the fatty acid biosynthesis and metabolism enrichment from the transcriptome analysis, ten 40-day-old bees from each cage were pooled as one sample, and three repeated samples per group were prepared and sent to Novogene Co., Ltd., Beijing, China, for LC/MS non-targeted lipidome analysis. The raw data of LC/MS was subjected to qualitative and quantitative analysis through blasting with the library of Lipidmaps and Lipiblast using Compound Discoverer (Version 3.1). The different lipid compounds between the two groups were screened based on the threshold of VIP > 1 (variable importance in the projection, VIP), FoldChange > 2 or FC < 0.5, and *p* < 0.05. These different lipid compounds were further subject to clustering (Python 3.5.0, R 3.4.3), KEGG classification (Python 3.5.0, R 4.0.3), and GSEA enrichment analysis (gsea 3.0).

### 2.4. RNA Extraction and cDNA Synthesis

TRizol (Invitrogen, Carlsbad, CA, USA) was used for honey bee RNA extraction, as described by the protocol. Hifair^®^ 1st Strand cDNA Synthesis SuperMix for qPCR (Yeasen Biotech Co., Ltd., Shanghai, China) was used for cDNA synthesis from the extracted RNA samples, as per the manual’s instructions. The synthesized cDNA was stored at −20 °C for use.

### 2.5. Fluorescent Real-Time Quantitative PCR (qPCR)

qPCR was used for verification of the transcriptome sequencing result and determination of the expression of fatty acid metabolism-related genes and the expression of royal jelly-secreting-related genes. The specific primers of these objective genes were designed by either using the online tools of Primer-Blast (https://www.ncbi.nlm.nih.gov/tools/primer-blast/index.cgi, accessed on 15 April 2025) or referring to previous reports. For detailed primer information, refer to the attached file (Supplementary Table S1). *β-actin* was used as a reference [40].

Several 10 µL reactions were set up with SYBR Green Master Mix (Yeasen Biotech Co., Ltd., Shanghai, China) 5.0 µL, forward primer 0.5 µL, reverse primer 0.5 µL, cDNA template 1.0 µL, and H_2_O 3.0 µL. An ABI QuantStudio 6 Flex System (Thermo Fisher Scientific, Waltham, MA, USA) was used to run the reaction, with the program as follows: denaturation at 95 °C for 3 min, and then 40 cycles of denaturation at 95 °C for 20 s, annealing at 60 °C for 20 s, and extension at 72 °C for 25 s, followed by a standard melting curve process.

### 2.6. Statistical Analysis

GraphPad Prism (Version 9.5, GraphPad, La Jolla, CA, USA) was used to analyze the experimental data and create figures. The log-rank (Mantel–Cox) test was used to analyze the difference in survival rate between the two groups. Student’s *t*-test with a post hoc Tukey’s honestly significant difference test (Tukey HSD) was used to evaluate the statistical significance of data on sugar solution consumed, qPCR verification of the transcriptome sequencing, fatty acid metabolism-related gene expression, honey bee head weight, and royal jelly secretion-related gene expression between the honey bees from the two groups. Presented data are means ± SEM, and *p* < 0.05 was considered statistically significant.

## 3. Results

### 3.1. High Colony Temperature Decreased the Survivorship of Adult Bees

**Figure 1:**
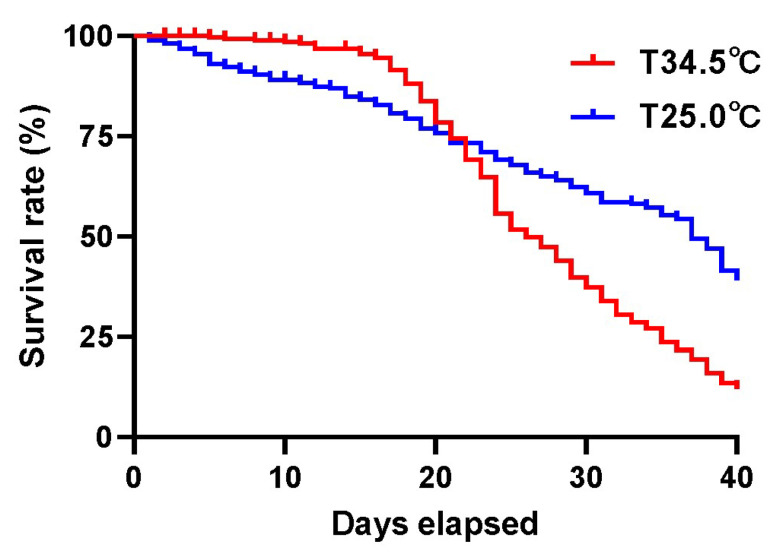
Survivorship of honey bees under colony temperature (34.5 °C) and room temperature (25.0 °C), respectively. Honey bees reared under room temperature have longer lifespans than honey bees reared under colony temperature (log-rank (Mantel–Cox) test, *χ* = 30.71, *p* < 0.0001).

Survival rate analysis showed that the honey bees reared at 34.5 °C had a high survival rate before 20 days of age, but died quickly after that. Instead, the honey bees reared at 25.0 °C maintained a relatively stable survivor trend from the test beginning to the end (Figure 1). In general, the honey bees kept at 25.0 °C had higher survivorship, with a median survival of 37 days, than the honey bees kept at 34.5 °C, with a median survival of 26 days (log-rank (Mantel–Cox) test, *χ* = 30.71, *p* < 0.0001). The high colony temperature in fact hurts the lifespan of adult worker bees.

### 3.2. High Temperature Increased the Sugar Consumption

**Figure 2:**
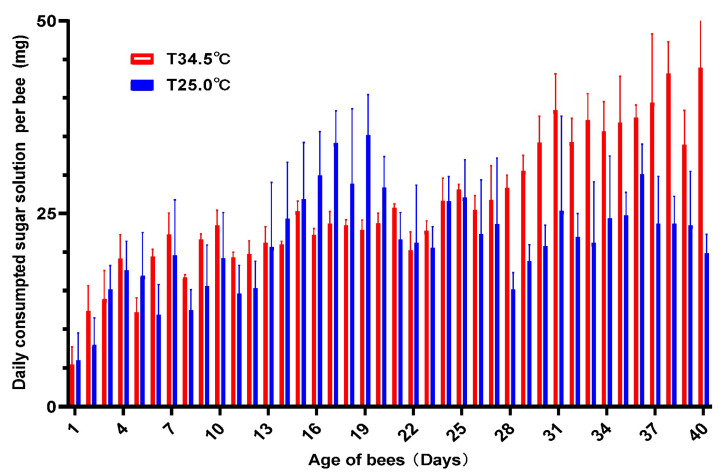
Daily consumed sugar solution between the honey bees reared at 34.5 °C and 25.0 °C. The average amount of sugar solution consumed per bee reared at 34.5 °C was significantly higher than that for bees reared at 25.0 °C (paired *t*-test, two-tailed, *t* = 3.542, *df* = 39, *p* = 0.0010).

Investigation of the daily consumed sugar solution showed that the average amount of daily consumed sugar solution was 21.24 mg for bees reared at 25.0 °C and 25.59 mg for those reared at 34.5 °C per bee. Honey bees reared at 34.5 °C consumed 4.52 mg more sugar solution daily than honey bees reared at 25.0 °C (Figure 2). Paired *t*-test analysis showed that the amount of daily consumed sugar solution between the two groups was significantly different (paired *t*-test, two-tailed, *t* = 3.542, *df* = 39, *p* = 0.0010). Honey bees reared at 34.5 °C consumed more sugar solution than those reared at 25.0 °C.

### 3.3. Transcriptome Sequencing

**Figure 3:**
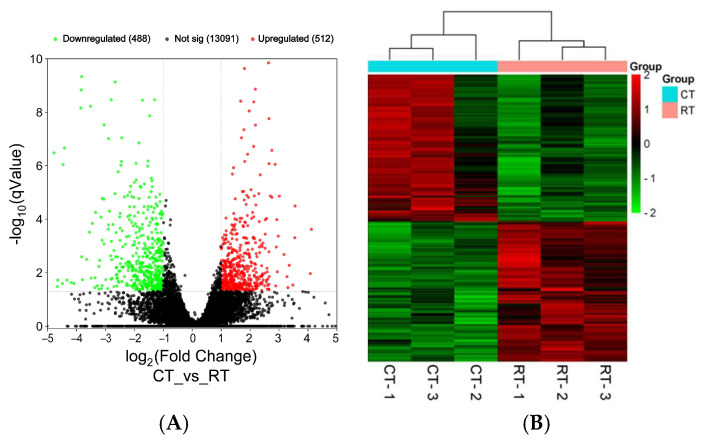
Volcano map (**A**) and clustering analysis (**B**) of the genes from the honey bees of the two temperature groups. CT represents the colony temperature of 34.5 °C; RT represents the room temperature of 25.0 °C. Red dots in A mean upregulated, green dots mean downregulated, and black dots mean not changed. In total, 512 upregulated genes and 488 downregulated genes in honey bees reared at 34.5 °C compared with honey bees reared at 25.0 °C were found from the 14,091 genes assembled. Clustering of the gene expression profile from all three samples of the two temperature groups showed good repeatability.

To investigate the background gene expression basis of the difference in longevity between the honey bees reared at the two temperatures, 40-day-old bees from the two groups were subject to transcriptome sequencing. The raw sequencing data of transcriptome sequencing were deposited under the BioProject of PRJNA1250038 in the Sequence Read Archives (SRAs) at the NCBI. The result showed that among the total 14,091 genes assembled, 512 upregulated genes and 488 downregulated genes were found between the honey bees reared at 34.5 °C and at 25.0 °C (Figure 3A). The gene expression profiles of the bees reared at 34.5 °C were clustered together, while those of the bees reared at 25.0 °C were clustered into another group, indicating the good repeatability of the transcriptome analysis (Figure 3B).

### 3.4. GO Enrichment

#### 3.4.1. Upregulated Genes Were Enriched in Fatty Acid Biosynthesis and Metabolism

**Figure 4:**
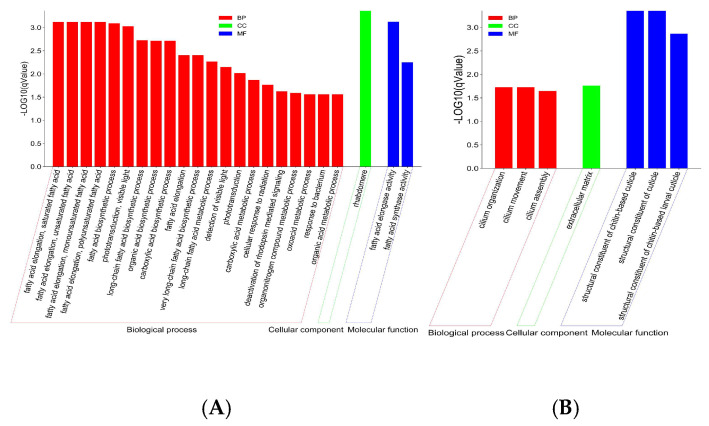
GO enrichment of the upregulated genes (**A**) and downregulated genes (**B**) in honey bees reared at 34.5 °C compared with honey bees reared at 25.0 °C. The upregulated genes were mainly enriched in fatty acid biosynthesis and metabolism-related items, and the downregulated genes were mainly enriched in cuticle structure and constituent-related items.

GO analysis showed that the 512 upregulated genes were significantly enriched in 24 GO items, including 21 items in biological processes such as fatty acid elongation, saturated fatty acid/unsaturated fatty acid/ monounsaturated fatty acid /polyunsaturated fatty acid (GO:0019367/GO:0019368/GO:0034625/GO:0034626), fatty acid biosynthetic process (GO:0006633), long-chain fatty acid biosynthetic process (GO:0042759), organic acid biosynthetic process (GO:0016053), carboxylic acid biosynthetic process (GO:0046394), fatty acid elongation (GO:0030497), very long-chain fatty acid biosynthetic process (GO:0042761), long-chain fatty acid metabolic process (GO:0001676), etc., 2 items in molecular functions, including fatty acid elongase activity (GO:0009922) and fatty acid synthase activity (GO:0004312), and 1 item of rhabdomere (GO:0016028) in cellular components (Figure 4A). Thereby, the honey bees reared at 34.5 °C significantly upregulated fatty acid biosynthesis and metabolism when compared to the bees reared at 25.0 °C.

#### 3.4.2. Downregulated Genes Were Enriched in the Structural Constituent of the Cuticle

The 488 downregulated genes were significantly enriched in seven items, including three items in the biological process of cilium organization (GO:0044782), cilium movement (GO:0003341), and cilium assembly (GO:0060271), three items in the molecular function of the structural constituents of the chitin-based cuticle (GO:0005214), the structural constituents of the cuticle (GO:0042302), and the structural constituents of the chitin-based larval cuticle (GO:0008010), and one item in the cellular components of the extracellular matrix (GO:0031012) (Figure 4B). Thereby, the honey bees reared at 34.5 °C had downregulated cuticle structure and constituent gene expression when compared with the bees reared at 25.0 °C.

### 3.5. KOG Enrichment

**Figure 5:**
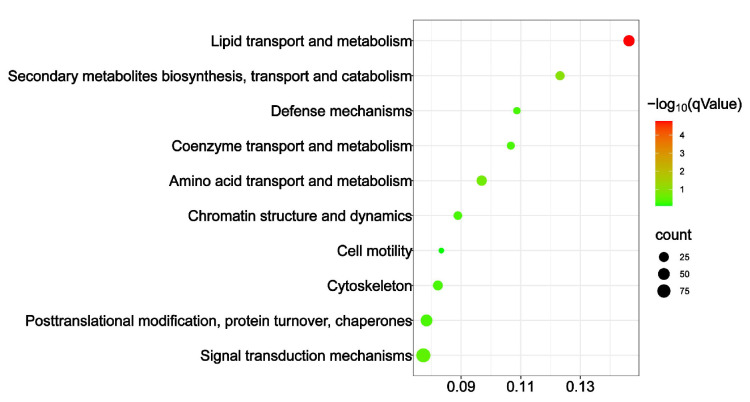
KOG enrichment of the DEG between the honey bees reared at 34.5 °C and 25.0 °C. Only lipid transport and metabolism were significantly enriched, implying that lipid transport and metabolism were altered in honey bees reared at different temperatures.

KOG analysis showed that only lipid transport and metabolism were significantly enriched, with qValue = 7.334 × 10^−7^. All the other functional classes were not statistically enriched (Figure 5). Such a result further verified that lipid transport and metabolism were the main differences between the honey bees reared at the two different temperatures.

### 3.6. qRT-PCR Verification

**Figure 6:**
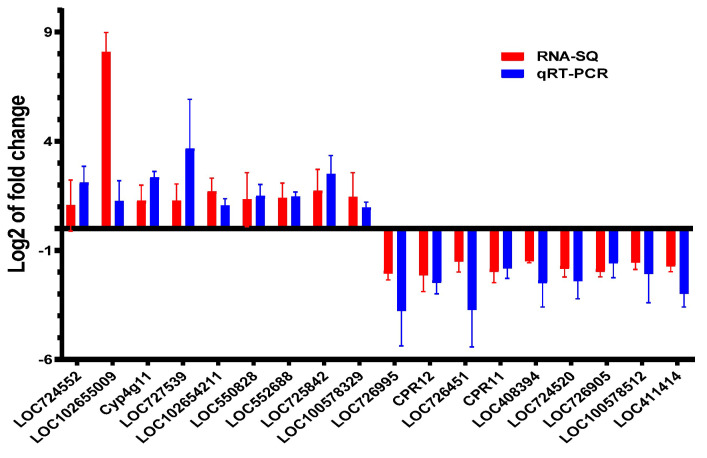
Verification of the transcriptome sequencing by qRT-PCR. The expression of six downregulated cuticle-related genes and nine upregulated fatty acid metabolism-related genes determined by transcriptome analysis were verified by using qRT-PCR. The result showed that their expressions coincided with their respective expression mode (upregulated or downregulated) determined by transcriptome analysis.

To verify the results of transcriptome sequencing, the expression of nine downregulated cuticle-related genes and nine upregulated fatty acid metabolism-related genes from the transcriptome analysis were selected for further investigation using qRT-PCR. The result showed that all the fatty acid metabolism-related genes were upregulated, and all the cuticle-related genes were downregulated using qRT-PCR, which was consistent with their respective expression mode determined by RNA sequencing (*t*-test, *t* = 1.173, *df* = 17, *p* = 0.2569) (Figure 6). Thereby, the result of RNA sequencing was reliable in this study.

### 3.7. Lipidomic Analysis

**Figure 7:**
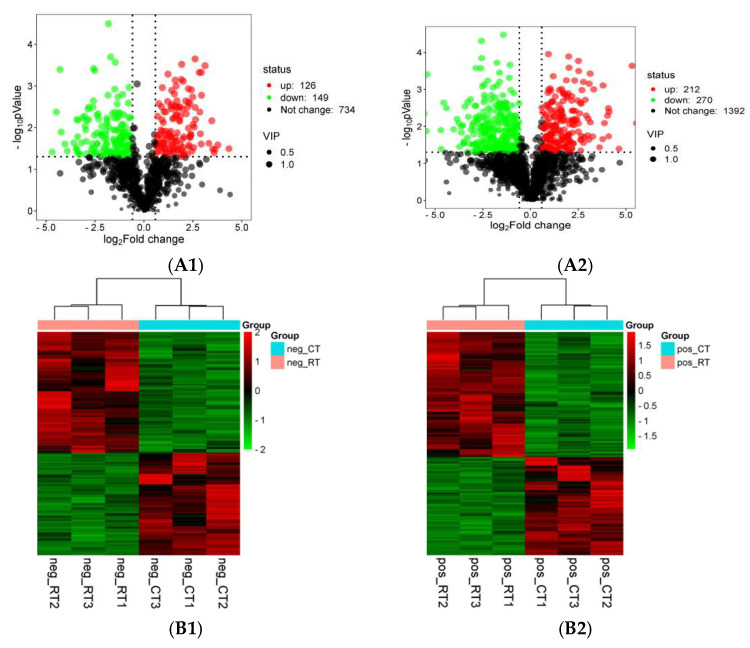

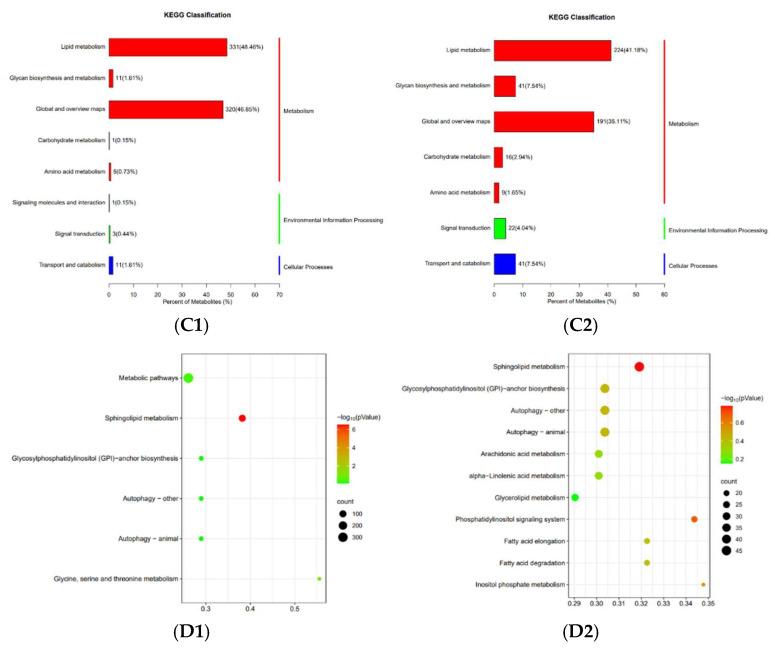
Lipidomic analysis between the honey bees reared at 34.5 °C and 25.0 °C. Volcano map of the lipid compounds in POS (**A1**) and NEG (**A2**) from the honey bees reared at 34.5 °C and 25.0 °C. Red dots mean upregulated, green dots mean downregulated, and the black dots mean not changed. Dot size means the value of VIP. The dashed line correspond to the value of FoldChange > 2 or FC < 0.5, and *p* < 0.05, which seperate the area of not changed, upregulated and downregulated, respectively, Clustering analysis of the different lipid compounds in POS (**B1**) and NEG (**B2**) between the honey bees reared at 34.5 °C and 25.0 °C. The red color represents the lipids that were upregulated; the green color represents the lipids that were downregulated. KEGG classification of the different lipid compounds in POS (**C1**) and NEG (**C2**) between the honey bees reared at 34.5 °C and 25.0 °C. The *X*-axis represents the percentage of lipid compounds enriched in specific pathways relative to all the lipid compounds annotated. The right *Y*-axis is the first-level classification of the KEGG pathway, and the left *Y*-axis is the second-level classification of the KEGG pathway. The majority of the different lipids’ compounds from both the POS and NEG parts were enriched in lipid metabolism and the global and overview maps. KEGG enrichment of the different lipid compounds in POS (**D1**) and NEG (**D2**) between the honey bees reared at 34.5 °C and 25.0 °C. The *X*-axis represents the ratio of the detected lipid compounds in the specific pathway to all the lipid compounds in the pathway. The size of the dots represents the amount of annotated lipid compounds in the pathway. The color of the dots represents the −log10 of the *p*-value, with red indicating significantly enriched and green not enriched. The sphingolipid metabolism pathway was the only pathway significantly enriched.

Fatty acid biosynthesis and metabolism were effectively enriched in the transcriptome analysis of the honey bees reared at different temperatures. To further validate this, the 40-day-old bees from the two temperature groups were sampled and subjected to lipidomic analysis. The result showed that 482 significantly different lipid compounds were identified from the 1874 compounds identified in total in POS lipids, among which 270 compounds were upregulated and 212 compounds were downregulated in the honey bees reared at 34.5 °C compared with the honey bees reared at 25.0 °C (Figure 7A1). In NEG lipids, 275 significantly different lipid compounds were identified from the 1009 compounds identified in total, among which 149 compounds were upregulated and 126 compounds were downregulated (Figure 7A2). Honey bees reared at different temperatures have significantly different lipid components.

Clustering analysis showed that the content and components of POS lipids from the three samples of honey bees reared at 34.5 °C shared similar profiles, and could be clustered into one branch, while the POS lipids from the three samples of honey bees reared at 25.0 °C shared similar profile too, and could be clustered into another branch (Figure 7B1). NEG lipids in the three samples of honey bees reared at 34.5 °C and 25.0 °C, respectively, displayed a similar result (Figure 7B2). Such a result showed the good repeatability of the lipidomic test.

KEGG classification of the different lipid compounds in POS showed that 48.46% of the metabolites were classified into lipid metabolism, and 46.85% of the metabolites were classified into global and overview maps at the second classification level under the same first classification level of metabolism (Figure 7C1). KEGG classification of the different lipid compounds in NEG showed a similar result (Figure 7C2). This validated that the lipid metabolism between the honey bees reared at 34.5 °C and 25.0 °C was significantly different.

KEGG enrichment was performed to determine the possible pathway of the different lipid compounds involved between honey bees reared at 34.5 °C and 25.0 °C. The result showed that only the pathway of sphingolipid metabolism was significantly enriched from the different lipid compounds in POS (Figure 7D1). The same sphingolipid metabolism pathway was enriched from the different lipid compounds in NEG too (Figure 7D2). Therefore, the alteration in fatty acid metabolism in honey bees reared at different temperatures was mainly enriched in the sphingolipid metabolism pathway.

### 3.8. Upregulated Fatty Acid Genes in Young Adult Bees Reared at 34.5 °C

**Figure 8:**
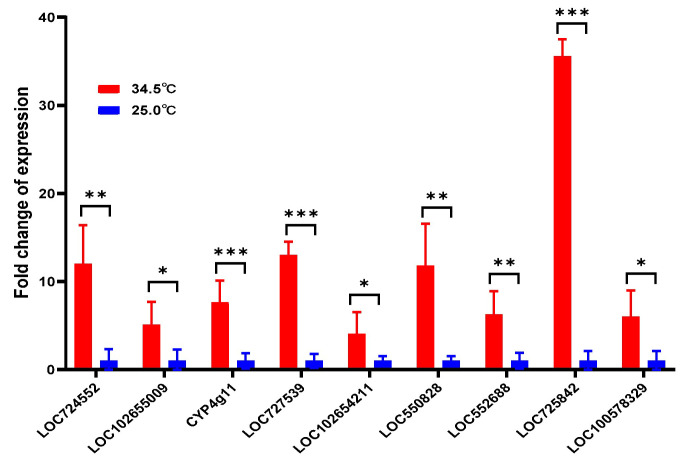
Relative expression of fatty acid metabolism-related genes in young adult bees of 5 days old reared at 34.5 °C and 25.0 °C, respectively. All 9 fatty acid metabolism-related genes were upregulated in 5-day-old adult bees reared at 34.5 °C compared to 25.0 °C. * means *p* < 0.05; ** means *p* < 0.01; *** means *p* < 0.001.

The expression of fatty acid metabolism-related genes in young adult bees of 5 days old was also investigated. The result showed that all the nine fatty acid metabolism-related genes were also upregulated in 5-day-old adult bees reared at 34.5 °C compared to the bees reared at 25.0 °C (*t*-test, for LOC724552, *t* = 5.368, *p* = 0.0010; for LOC102655009, *t* = 2.789, *p* = 0.0385; for *CYP4g11*, *t* = 5.543, *p* = 0.0008; for LOC727539, *t* = 13.58, *p* < 0.0001; for LOC102654211, *t* = 2.676, *p* = 0.0281; for LOC550828, *t* = 4.446, *p* = 0.0030; for LOC552688, *t* = 3.768, *p* = 0.0093; for LOC725842, *t* = 36.73, *p* < 0.0001; for LOC100578329, *t* = 3.126, *p* = 0.0261) (Figure 8). Such results implied that the upregulating effect of high temperature on honey bees’ fatty acid metabolism-related genes was independent of the honey bees’ age.

### 3.9. Heavier Head Weight in Young Adult Bees Reared at 34.5 °C

**Figure 9:**
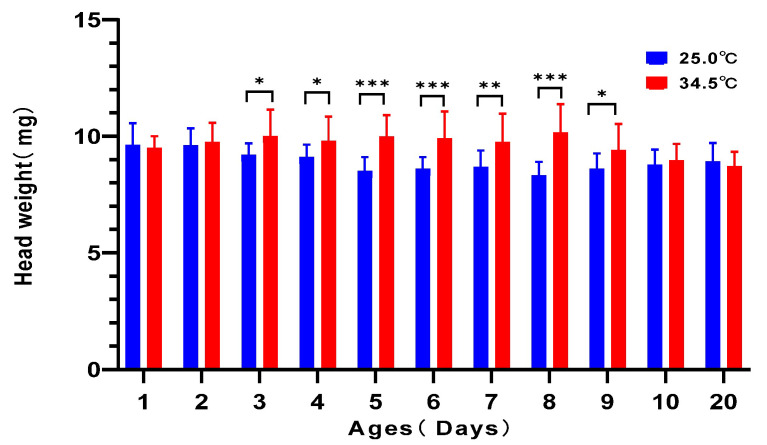
The head weight of young adult bees reared at 34.5 °C and 25.0 °C, respectively. The honey bees from 3 days old to 9 days old reared under colony temperature have a heavier head weight than the bees reared under room temperature. * means *p* < 0.05; ** means *p* < 0.01; *** means *p* < 0.001.

The head is the position where the hypopharyngeal gland is located. Its weight is related to the development and physiological stage of the hypopharyngeal gland. The newly emerged bees quickly developed their hypopharyngeal gland by consuming honey and pollen, so that they could undertake the brood-nursing task in the colony. By analyzing the head weight of the young adult bees reared under the two different temperatures, we found that the head weight of honey bees from 3 days old to 9 days old kept at 34.5 °C was significantly higher than that of corresponding-age bees kept at 25.0 °C (*t*-test, for 3-day-old bees: *t* = 2.541, *df* = 30, *p* = 0.0165; for 4-day-old bees: *t* = 2.323, *df* = 30, *p* = 0.0272; for 5-day-old bees: *t* = 5.287, *df* = 30, *p* < 0.0001; for 6-day-old bees: *t* = 24.005, *df* = 30, *p* = 0.0004; for 7-day-old bees: *t* = 3.000, *df* = 30, *p* = 0.0054; for 8-day-old bees: *t* = 5.300, *df* = 30, *p* < 0.0001; for 9-day-old bees: *t* = 2.225, *df* = 26, *p* = 0.0097). The head weight of 1-, 2-, 10-, and 20-day-old adult bees between the two temperature groups was not different (for 1-day-old bees: *t* = 0.4355, *df* = 30, *p* = 0.6663; for 2-day-old bees: *t* = 0.4588, *df* = 30, *p* = 0.6497; for 10-day-old bees: *t* = 0.7501, *df* = 30, *p* = 0.4591; for 20-day-old bees: *t* = 0.7656, *df* = 30, *p* = 0.4499) (Figure 9). Such results showed that the newly emerged adult bees kept at 34.5 °C increased their head weight quicker than those kept at 25.0 °C.

### 3.10. Upregulated Royal Jelly Secretion-Related Genes in Young Adult Bees Reared at 34.5 °C

**Figure 10:**
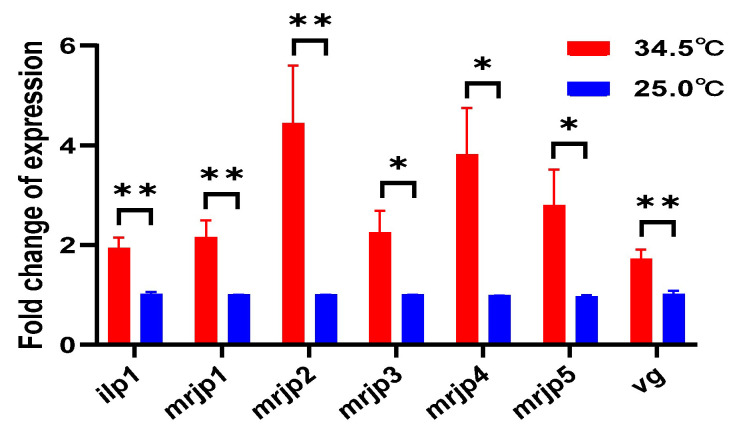
Expression of royal jelly secretion-related genes in 5-day-old adult bees reared under colony temperature (34.5 °C) and room temperature (25.0 °C), respectively. All these genes’ expression was increased in the bees reared under colony temperature compared to the bees reared under room temperature. * means *p* < 0.05; ** means *p* < 0.01.

To further determine the activity of the hypopharyngeal gland, the 5-day-old adult bees were collected to investigate the gene expression of mrjp1–5 and two nutrient-related genes of *ilp1* and *vg*. The result showed that the expression of all the five *mrjps*, *ilp1*, and *vg* were significantly upregulated in 5-day-old adult bees reared at 34.5 °C compared to those kept at 25.0 °C (*t*-test, for *ilp1*, *t* = 3.960, *df* = 12, *p* = 0.0027; for *mrjp1*, *t* = 3.247, *df* = 12, *p* = 0.0070; for *mrjp2*, *t* = 3.819, *df* = 11, *p* = 0.0028; for *mrjp3*, *t* = 2.790, *df* = 12, *p* = 0.0164; for *mrjp4*, *t* = 2.502, *df* = 10, *p* = 0.0314; for *mrjp5*, *t* = 2.504, *df* = 12, *p* = 0.0277; for *vg*, *t* = 3.470, *df* = 12, *p* = 0.0046) (Figure 10). These results validated the active royal jelly secretion of the hypopharyngeal gland in honey bees kept at 34.5 °C.

### 3.11. Developed Hypopharyngeal Gland in Young Adult Bees Reared at 34.5 °C

**Figure 11:**
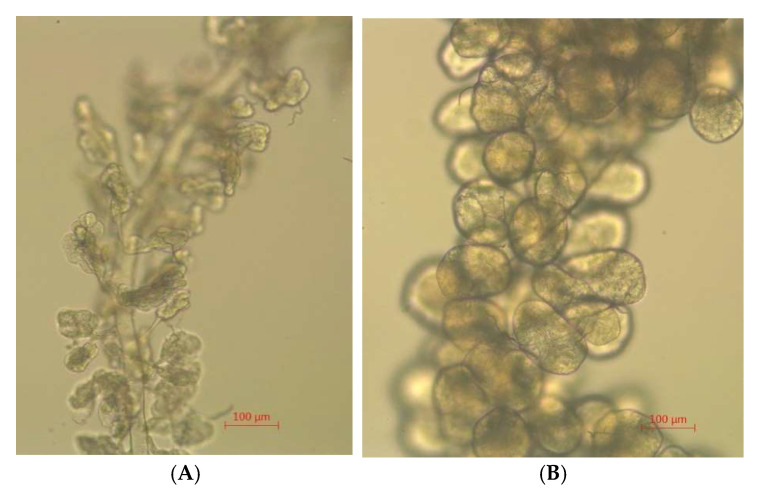
Morphology of hypopharyngeal gland acini of 5-day-old adult bees reared under colony temperature (34.5 °C) and room temperature (25.0 °C), respectively. (**A**) Honey bees reared under room temperature (25.0 °C). (**B**) Honey bees reared under colony temperature (34.5 °C). Five-day-old adult bees reared under colony temperature have more developed hypopharyngeal gland acini.

The size and morphology of hypopharyngeal gland acini are related to their activity. By observing the hypopharyngeal gland under a microscope, we found that all the acini from the 5-day-old honey bees reared at 34.5 °C were round and full of content. Most of the acini from the 5-day-old adult bees kept at 25.0 °C were collapsed and had no content in the cells. Thereby, the hypopharyngeal glands from the honey bees reared at 34.5 °C were more developed than those of the honey bees reared at 25.0 °C (Figure 11).

## 4. Discussion

Temperature is one of the critical environmental factors determining the survival of animals. Honey bees have evolved a trait of a relatively high temperature requirement of around 34.5 °C for the normal development of larvae, and the colony correspondingly maintains this temperature throughout the whole brood-rearing season. But in the non-brood-rearing stage, the temperature of the colony changes accordingly with the ambient temperature. Rearing more broods certainly increases the future competition and survivability of the colony, and the potential bee products for the beekeeper. Therefore, the positive aspects of rearing the brood colony at the high colony temperature have been considered most often. However, 34.5 °C is a relatively high external temperature for most animals [41]. A honey bee colony normally has a reproduction season that lasts over six months, and the normal lifespan of worker bees is less than 50 days [42], which means that the adult bees in the colony inevitably suffer from long-term thermal stress. However, what the specific effect of the high temperature on adult bees is, and the adult bees’ adaption to it, have not been investigated in previous works. The background biological sense of maintaining such a high colony temperature is not clear either. Winter bees, with a completely different physiological state with summer bees, and never facing the high colony temperature of 34.5 °C, were therefore not included in this study. Only newly emerged adult bees in summer were reared in cages under a colony temperature of 34.5 °C and room temperature of 25.0 °C, respectively. Their survival rate, the morphology of the hypopharyngeal gland, nutrient gene expression, the transcriptome, and the lipidome were investigated in detail. The result showed that the high temperature could impair the longevity of adult bees, alter the lipid metabolism, and quicken the process of newly emerged bees becoming nurses, which increased the colony nurturing capability. This finding uncovered the negative effect of a colony temperature of 34.5 °C on adult bees, and its colony-level beneficial significance, which provides a basis for honey bee biology research and related applications.

Thermal stress is an important and universal modulator of longevity and aging both in poikilotherm and homeotherm animals [7,10,11,12]. High temperature has adverse effects on aging and longevity [8,9,16,19], which were verified in multiple animals with different evolution levels, including *monogonont rotifer*, *Brachionus manjavacas* [17], *Caenorhabditis elegans* [13,14,15], aquatic animals [16,43], and mammal animal [18]. The short-lived adult bees caused by the high temperature in this study were like these reports (Figure 1). The traditional rate-of-living theory states that high temperatures increase chemical reaction rates, thus speeding up the aging process [44]. The consumption of sugar solution by honey bees kept at 34.5 °C was significantly higher than that of bees kept at 25 °C (Figure 2), which partly coincides with this theory.

In addition, previous reports showed that the modulation of temperature on lifespan is actively regulated by genes, including those encoding thermosensitive TRP channels [22,23,24]. Changes in the genes’ expression were the response of honey bees to a variety of external factors. Given the significant difference in survival rate between the two temperature groups, to determine the background modulating genes of temperature on honey bees’ longevity, 40-day-old bees from the two different temperature groups were collected for transcriptome analysis in this study. The result showed that the fatty acid biosynthesis and metabolism-related genes were upregulated in bees from the 34.5 °C group. Previous reports verified that fatty acid metabolism could regulate longevity [16,45]. Fatty acid is the main component of cell membranes and mitochondrial membranes [46]. Any change in fatty acid metabolism could affect the structure and function of the membrane and the cell’s destination or longevity at the organismal level [47]. Moreover, the cell’s normal metabolism produces free radicals, which cause lipid peroxidation and shorten the lifespan in Drosophila [45]. Any free radical removal mechanism such as superoxide dismutase and phospholipase A2 activity could change the aging process [20]. Furthermore, thermosensitive TRP channels were taken as modulators of organism longevity [22,23,24]. Lipids have been reported to regulate the activity of TRP ion channels, thus affecting the lifespan of target organisms [48,49,50,51,52,53]. Fatty acid metabolism alteration was thereby one of the critical mechanisms responsible for longevity regulation.

Contrary to the upregulation of fatty acid metabolism-related genes, the downregulated of wax synthesis- and cuticle function-related genes were enriched in bees kept at 34.5 °C (Figure 4). The cuticle is an interface in insects that regulates temperature and moisture evaporation. Wax synthesis and cuticle structure could affect the permeability, which alters the body temperature and water balance. Decreasing wax synthesis and cuticle-related gene expression could increase cuticle permeability and moisture evaporation for maintaining a normal body temperature. Moreover, a previous report showed that one of the fatty acid metabolism-related genes, CYP4G11, also enriched in this study, could be taken as an oxidative decarbonylase to metabolize shorter-chain aldehydes to produce cuticular hydrocarbon for preventing desiccation or comb wax production [54]. Thereby, fatty acid metabolism is also involved in wax synthesis and cuticle structure and functions.

Fatty acid metabolism was significantly enriched from the differently expressed genes between the honey bees reared at the two temperatures at the transcriptome level (Figure 3). The lipidomic analysis further validated that the lipid compounds in both the POS and NEG part from the honey bees reared at 34.5 °C were different from those in the honey bees reared at 25.0 °C (Figure 7A1,A2,B1,B2). The majority of these different lipid compounds were classified into the lipid metabolism and the global and overview maps at the second classification level under the same first classification level of metabolism in the KEGG classification analysis (Figure 7C1,C2). Such a result further verified the alteration in lipid metabolism in honey bees when reared at different temperatures.

The KEGG analysis showed that only the sphingolipid metabolism pathway was significantly enriched from the different lipid compounds between honey bees reared at 34.5 °C and 25.0 °C (Figure 7D1,D2). Sphingolipids are a class of bioactive complex lipids that have been closely associated with aging and aging-related diseases through controlling lysosomal homeostasis and function [55]. Moreover, sphingolipids are one of the lipids that compose mitochondria. Changes in sphingolipids have been implicated as being an essential step in mitochondria-driven cell death [56,57]. Sphingolipid synthetic pathways are also the major regulators of lipid homeostasis [58]. Previous reports showed that reducing sphingolipid biosynthesis could promote longevity in several model organisms [59,60,61,62,63,64,65]. The shortened lifespan of honey bees reared at 34.5 °C compared with honey bees reared at 25.0 °C in this study might be caused by the upregulation of sphingolipids. Sphingolipids could be taken as a marker of honey bees’ longevity.

In this study, the relatively low temperature of 25 °C extended the lifespan of adult bees overall (Figure 1). However, the survival curve did not consistently show the trend of a high survival rate of honey bees reared at 25 °C. Instead, the bees less than 20 days old reared at 34.5 °C showed a higher survival rate than the bees kept at 25.0 °C. Only over-20-day-old bees reared at 34.5 °C exhibited a higher death rate than bees reared at 25.0 °C (Figure 1). The colony maintains a high and stable colony temperature of 34.5 °C, fulfilling the brood’s optimal development requirement. Our result implied that the young adult bees, like the larvae in the colony, still need a high temperature of 34.5 °C to maintain their normal development.

Referring to the upregulated fatty acid metabolism-related genes in honey bees of 40 days old reared at 34.5 °C in this study (Figure 4, Figure 5 and Figure 6), their expression in young adult bees of 5 days old using qRT-PCR methods was verified to be upregulated too (Figure 8). Such a result showed that the upregulation of fatty acid metabolism-related genes in honey bees reared at 34.5 °C is independent of their age. The honey bee is a eusocial insect. The longevity of worker bees is normally less than 50 days [42]. The survival of a colony is indispensable to the continuous supplying of newly emerged bees. The newly emerged bees result from the effective larvae nursing capability in the colony. Royal jelly is a nutritious substance secreted by the hypopharyngeal gland located in the head of nurse bees. Young larvae less than 3 days old completely rely on the royal jelly. The number of nurse bees and their royal jelly secretive activity fundamentally determines the scale of larvae rearing in the colony. Royal jelly contains around 3–6% lipids, including fatty acids [66,67,68,69]. The high expression of fatty acid-related genes might be the molecular basis of nurse bees secreting royal jelly. The heavier head weight, higher mrjp1–5 expression, and more developed hypopharyngeal gland in honey bees reared at 34.5 °C compared to the bees reared at 25.0 °C further verified the connection between the upregulated expression of fatty acid-related genes and the nurse bees’ royal jelly secreting activity (Figure 9, Figure 10 and Figure 11) [70]. Such results displayed that the newly emerged bees at a colony temperature of 34.5 °C had a quick development process to become nurse bees, maximizing the colony larvae nursing capability [71], which increased the colony survivability. Therefore, the relatively high colony temperature has a positive significance at the colony level, regardless of the cost of a shortened lifespan of adult bees at the individual level.

## 5. Conclusions

High temperatures normally shorten the longevity of organisms. However, honey bees have evolved to maintain a high colony temperature of 34.5 °C in brood-rearing seasons. Such temperature is optimal for larvae development, increasing the colony reproduction efficiency. In this study, the colony temperature of 34.5 °C was validated to have an overall negative effect on honey bee’s longevity when compared with 25.0 °C, which is caused by the increase in the expression of fatty acid metabolism-related genes and the alteration in the pathway of sphingolipid metabolism. Such upregulation of fatty acid metabolism-related genes at 34.5 °C was also found in young adult bees, which was proved to be related to nurse bee development and associated royal jelly secretion. Except for the well-known temperature requirement of the larvae and pupae development of 34.5 °C, this study validated that young adult bees still require such high temperature to maintain their normal development. The upregulation of fatty acid metabolism-related genes in young adult bees at 34.5 °C was positively related to their royal jelly secretion activity and the whole colony nursing capability, which determined the development speed, scale, and survivability of the colony. The colony temperature of 34.5 °C is a trade-off made by honey bees between the shortened lifespan of individual adult bees and increased colony survivability.

## Data Availability

The raw data of transcriptome sequencing were deposited under the BioProject of PRJNA1250038 with the accession No. of SAMN47928621, SAMN47928622, SAMN47928623, SAMN47928624, SAMN47928625, and SAMN47928626, in the Sequence Read Archives (SRAs) at the NCBI. The other data presented in this study are available on request from the corresponding author.

## Supplementary Materials

The following supporting information can be downloaded at https://www.mdpi.com/article/10.3390/insects16060558/s1: Table S1: Primers used for validation the result of RNA sequencing and determination of the gene expression in qRT-PCR.
